# Supplementary material for: The interactome of intact mitochondria by cross-linking mass spectrometry provides evidence for coexisting respiratory supercomplexes
Source: Mol Cell Proteomics. 2017 Dec 8;17(2):216–32. doi: 10.1074/mcp.RA117.000470 (PMC5795388; doi:10.1074/mcp.RA117.000470)
Supplement: Supplemental Data [file supp_RA117.000470_133922_0_supp_23978_fzffwf.zip › spectra_annotation/mito_DR_spectra_annotation/411-1-5-1-1-1.pdf]

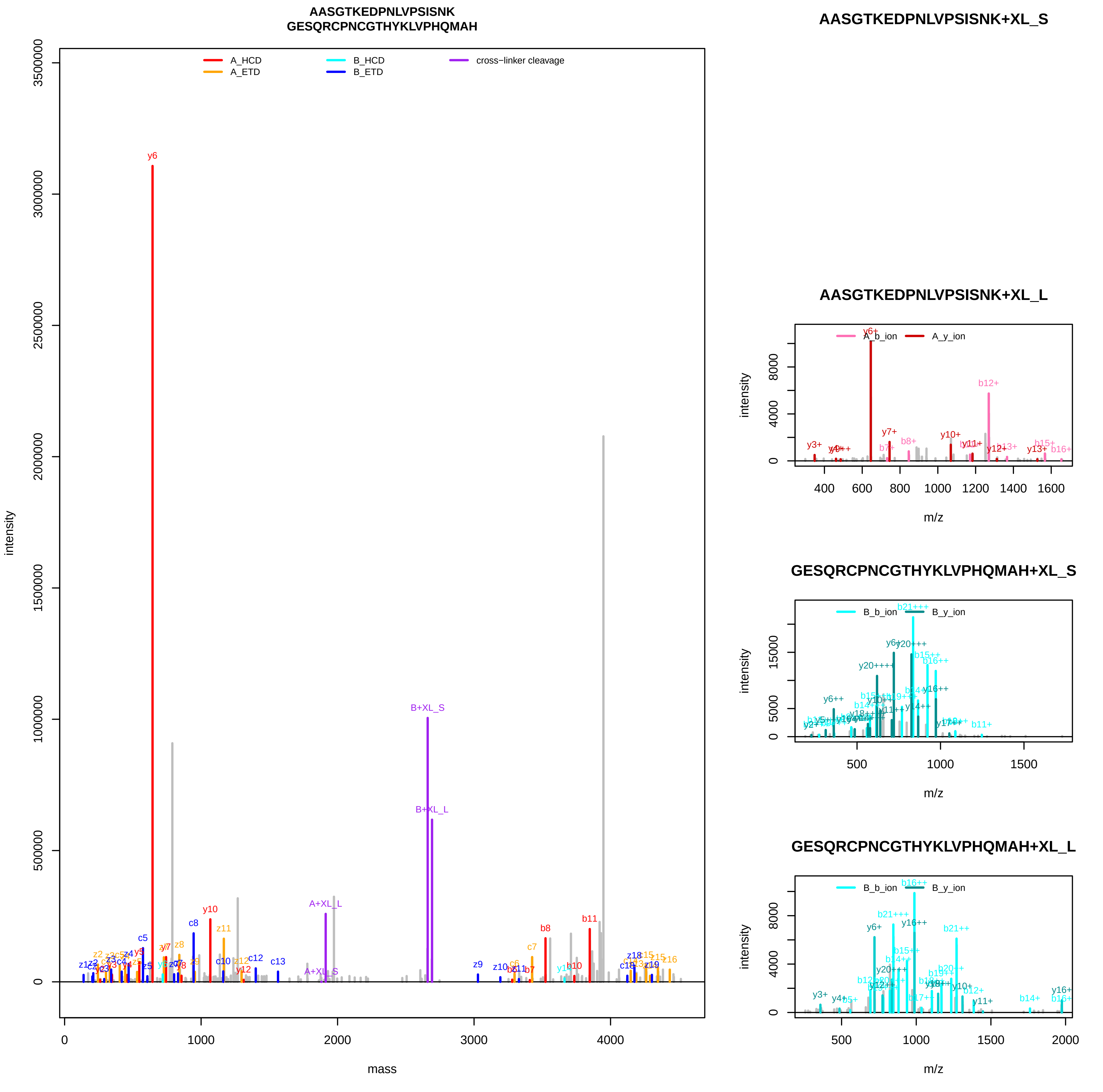

AASGTKEDPNLVPSISNK
GESQRCPNCGTHYKLVPHQMAH
A_HCD
A_ETD
B_HCD
B_ETD
cross−linker cleavage
intensity
mass
0
500000
1000000
1500000
2000000
2500000
3000000
3500000
1000
2000
3000
4000
y6
B+XL_S
B+XL_L
A+XL_L
A+XL_S
y10
b8
b11
b10
c8
c5
z11
c7
c10
c12
c13
z9
z10
c6
b7
z18
z16
AASGTKEDPNLVPSISNK+XL_S
AASGTKEDPNLVPSISNK+XL_L
A_b_ion
A_y_ion
y6+
b12+
y7+
y10+
b8+
y3+
b15+
b16+
y13+
y12+
b7+
m/z
400
600
800
1000
1200
1400
1600
8000
4000
GESQRCPNCGTHYKLVPHQMAH+XL_S
B_b_ion
B_y_ion
b21+++
y6+
y20+++
b15++
b16++
y20++++
y16++
y14++
y6++
y17++
b11+
500
1000
1500
15000
5000
GESQRCPNCGTHYKLVPHQMAH+XL_L
b16++
b21+++
y16++
b21++
y20+++
y3+
y4+
b5+
y11+
b14+
y16+
b16+
2000
